# Single photon emission computed tomography (SPECT) or positron emission tomography (PET) imaging for radiotherapy planning in patients with lung cancer: a meta-analysis

**DOI:** 10.1038/s41598-020-71445-5

**Published:** 2020-09-10

**Authors:** Soo Jin Lee, Hae Jin Park

**Affiliations:** 1 Department of Nuclear Medicine, Hanyang University Medical Center, 222-1 Wangsimni-ro, Seongdong-gu, Seoul, 04763 South Korea; 2 Department of Radiation Oncology, Hanyang University College of Medicine, 222-1 Wangsimni-ro, Seongdong-gu, Seoul, 04763 South Korea

**Keywords:** Biological techniques, Biotechnology, Cancer, Oncology

## Abstract

Functional imaging modalities enable practitioners to identify functional lung regions. This analysis evaluated the feasibility of nuclear medicine imaging to avoid doses to the functional lung in radiotherapy (RT) planning for patients with lung cancer. This systematic review and meta-analysis was carried out according to PRISMA-P guidelines. A search of EMBASE and PubMed for studies published throughout the last 20 years was performed using the following search criteria: (a) ‘lung cancer’ or ‘lung malignancy’ and (b) ‘radiotherapy’ or ‘radiation therapy’ or ‘RT planning’ and (c) ‘SPECT’ or ‘single positron emission computed tomography’ or ‘functional image.’ The analyzed planning parameters were the volumes of the normal lung that have received ≥ 10 Gy and ≥ 20 Gy of radiation (V10 and V20, respectively) and the mean lung dose (MLD). We compared the planning parameters obtained from anatomical RT planning and functional RT planning using perfusion or ventilation imaging (‘V10, V20 or MLD’ in anatomical plan vs. ‘fV10, fV20 or fMLD’ in functional plan). A total of 309 patients with 344 RT plan sets from 15 publications (11 perfusion SPECT, 2 ventilation SPECT, and 1 SPECT and 1 PET with both perfusion and ventilation) were included in the meta-analysis. The standard mean differences in planning parameters in functional plans using nuclear imaging were significantly reduced compared to those of anatomical plans (*P* < 0.01 for all): − 0.42 (95% confidence interval (CI) − 0.78 to − 0.07) for ‘V10 vs*.* fV10′, − 0.41 (95% CI − 0.64 to − 0.17) for ‘V20 vs*.* fV20′, and − 0.24 (95% CI − 0.45 to − 0.03) for ‘MLD *vs.* fMLD’. In subgroup analysis, the functional plan using perfusion was significantly lower than the anatomical plan in all planning parameters, but there was no significant difference for ventilation. RT planning with nuclear functional lung imaging has potential to reduce radiation-induced lung injury. Perfusion imaging seems to be more promising than ventilation imaging for all planning parameters. There were not enough studies using ventilation imaging to determine what the effect is on the lung plan parameters.

## Introduction

Radiotherapy (RT) plays a critical role in the management of early or locally advanced stage lung cancer. RT requires adequate coverage of the gross lung tumor as well as adjacent micrometastases for tumor control and sparing of the critical organs including the spinal cord and esophagus to minimize complications. Although enormous advances in RT planning and delivery techniques have improved loco-regional control and survivals in the past decades, radiation-induced lung injury still remains a common and possibly fatal dose-limiting toxicity that occurs in approximately 30% of lung cancer patients treated with RT^1^.

Dose–volume histogram (DVH) is a plot of a cumulative dose–volume frequency distribution, which graphically summarizes the simulated radiation distribution within a volume of interest of a patient that would result from a proposed radiation treatment plan based on computed tomography (CT) imaging^2^. Representative parameters, such as the mean lung dose (MLD) and the normal lung that has received ≥ 20 Gy of radiation (V20), has been important determinants of radiation-induced lung injury by assuming that all lung subvolumes are equally functioning. However, recent studies have shown that these parameters may not be sufficient to predict the risk of lung toxicity^3,4^. Since lung cancer is common especially in patients with respiratory comorbidities, such as chronic obstructive pulmonary disease (COPD) or emphysema related to long-standing smoking or interstitial lung disease, their lung tissue function may be spatially heterogeneous^5^.

Local lung function and distribution have been attempted to be quantitatively visualized in a quantitative way with various functional imaging modalities such as single photon emission computed tomography (SPECT), positron emission tomography (PET), CT ventilation with or without-dimensional (4D) technique, and hyperpolarized helium or xenon magnetic resonance imaging (MRI). Among functional lung images used to RT planning, SPECT images provided a unique quantitative 3-dimensional map of the distribution of functioning pulmonary vascular/alveolar subunits for the first time^6^. SPECT is a representative modality to design RT field and to provide a direct incidental irradiation away from volumes of well-functioning lung. Radiation oncologists have information on functional lung regions, and try to avoid irradiation to highly functioning lung tissue and ultimately to reduce pulmonary toxicity. Functional lung avoidance RT prioritizes delivery of high radiation dose to the lung cancer while minimizing RT dose to uninvolved and functional lung, through treatment planning system optimization algorithms^7^. Dose–functional histogram (DFH) is an emerging concept calculating the dose distribution throughout the functioning and non-functioning lung regions defined by functional imaging, compared to DVH. There is growing evidence that dose-volume parameters obtained DFH using functional imaging is a better predictor of radiation-induced lung injury (RILI) than those obtained DVH alone^3,4^.

A recent meta-analysis by Bucknell et al. suggested that functional lung imaging may have potential utility in RT planning and delivery^8^. However, they included all of the modalities such as SPECT, PET, and 4D-CT. Currently the clinical standard for functional lung imaging is the nuclear imaging of perfusion. CT ventilation imaging is a new modality that offers a higher resolution images compared with nuclear ventilation images and prove more convenient for patients undergoing RT planning. However, it showed a weak correlation with perfusion and ventilation SPECT on voxel-wise analysis, thus it needs to be validated against nuclear imaging^9^. In this study, we evaluated the feasibility of accurate prediction with nuclear medicine imaging in functional lung avoidance RT planning.

## Methods

A systematic review was performed using structured search terms following the Preferred Reporting Items for Systematic Reviews and Meta-Analyses (PRISMA-P) guidelines; the PRISMA-P checklist is presented in Supplemental Table 1^10^. Our research questions regarding the patients, intervention, comparison, outcome and study design (PICOS) approach are described in Table 1.

**Table 1:** PICOS table for study question.

	Contents
Patients	Lung cancer patients
Intervention	Functional plan using nuclear medicine imaging for radiation therapy
Comparison	Mean difference in V10, V20 or MLD between anatomical plan and functional plan using nuclear medicine imaging including ‘SPECT or PET’ of ‘perfusion or ventilation’ Functional plan between perfusion and ventilation images
Outcome	Functional plan better than anatomical plan for V10, V20 or MLD Which one is better: perfusion vs. ventilation?
Study	Retrospective or prospective study quantified human studies

V10 and V20 normal lung that has received ≥ 10 Gy and ≥ 20 Gy of radiation.

*MLD* mean lung dose, *SPECT* single positron emission computed tomography, *PET* positron emission tomography.

### Search strategy and study selection

Two authors (S. J. Lee and H. J. Park) performed a comprehensive computer literature search of two databases (EMBASE and PubMed) in March 2019 to identify relevant published studies from 2000 to 2019. The start date of 2000 was chosen to search the literature from the past 20 years. The studies identified from the literature search were evaluated for duplicates, and then full-text assessments were independently performed by two authors into four indications of the eligibility of an article, based on title and abstract screening. Many studies were not relevant to our study and were eliminated.

The following search criteria were used: (‘lung cancer’ or ‘lung malignancy’) and (‘radiotherapy’ or ‘radiation therapy’ or ‘RT planning’) and (‘SPECT’ or ‘single positron emission computed tomography’ or ‘functional image’). All searches were limited to human studies and English language. We did not limit the types of publication or numbers of included patients per study. The inclusion criteria for the relevant studies were as follows:

1. functional images (‘SPECT or PET’ of ‘perfusion or ventilation’) had been used to plan the radiation therapy in lung cancer;
2. a radiation therapy plan using CT (anatomical plan) had been used as the reference standard;
3. planning parameters [the volume of the normal lung that has received ≥ 10 Gy of radiation (V10), ≥ 20 Gy of radiation (V20), or MLD in RT plan using CT; fV10, fV20 or fMLD in RT plan using SPECT or PET images] in plans using anatomical and functional images had been presented.

The studies were excluded if the mean ± standard deviation (SD) or median with lowest and highest values in planning parameters had not been representative. The publications such as review articles, conference papers, or letters, which do not contain the original data, were excluded. When the data were published in more than one article, the latest one was included.

### Data extraction

Two reviewers independently extracted data from each article and recorded them on a standardized form. The agreement was 75%. Any disagreement in data extraction was resolved by consensus. The following data were extracted from each study:

1. first author name, article type, year of publication, age of patients, number of patients, and study design (prospective or retrospective)
2. technical characteristics of ‘SPECT or PET’ of ‘perfusion or ventilation’
3. detailed information of radiation therapy plan
4. raw data as well as means ± SD or medians with lowest and highest values of V10, V20, or MLD between two plans.

### Quality assessment

A quality assessment was developed based on the appraisal standard of the Newcastle–Ottawa scale (NOS). NOS score of ≥ 6 were assigned as high-quality studies. Consensus was reached by discussion when disparity occurred.

### Statistical analysis

All analyses and corresponding plots were performed using the statistical software R, version 3.6.1 (R Foundation for Statistical Computing)^11^, and the “meta”^12^ and “metaphor” packages^13^. A meta-analysis was performed for the differences in V10, V20, and MLD between anatomical and functional plans. Not all studies provided the mean values and SDs. If raw data were not available and median values with confidence intervals were provided, these were calculated using the methods described^14^. Heterogeneity was assessed by the likelihood ratio I^2^ index, which was considered high when greater than 50%. The meta-analysis was performed for the standard mean differences (SMD) between means using inverse variance and random effects. Publication bias was assessed by Funnel plots and trim-and-fill analysis.

## Results

### Study selection

**Figure 1:**
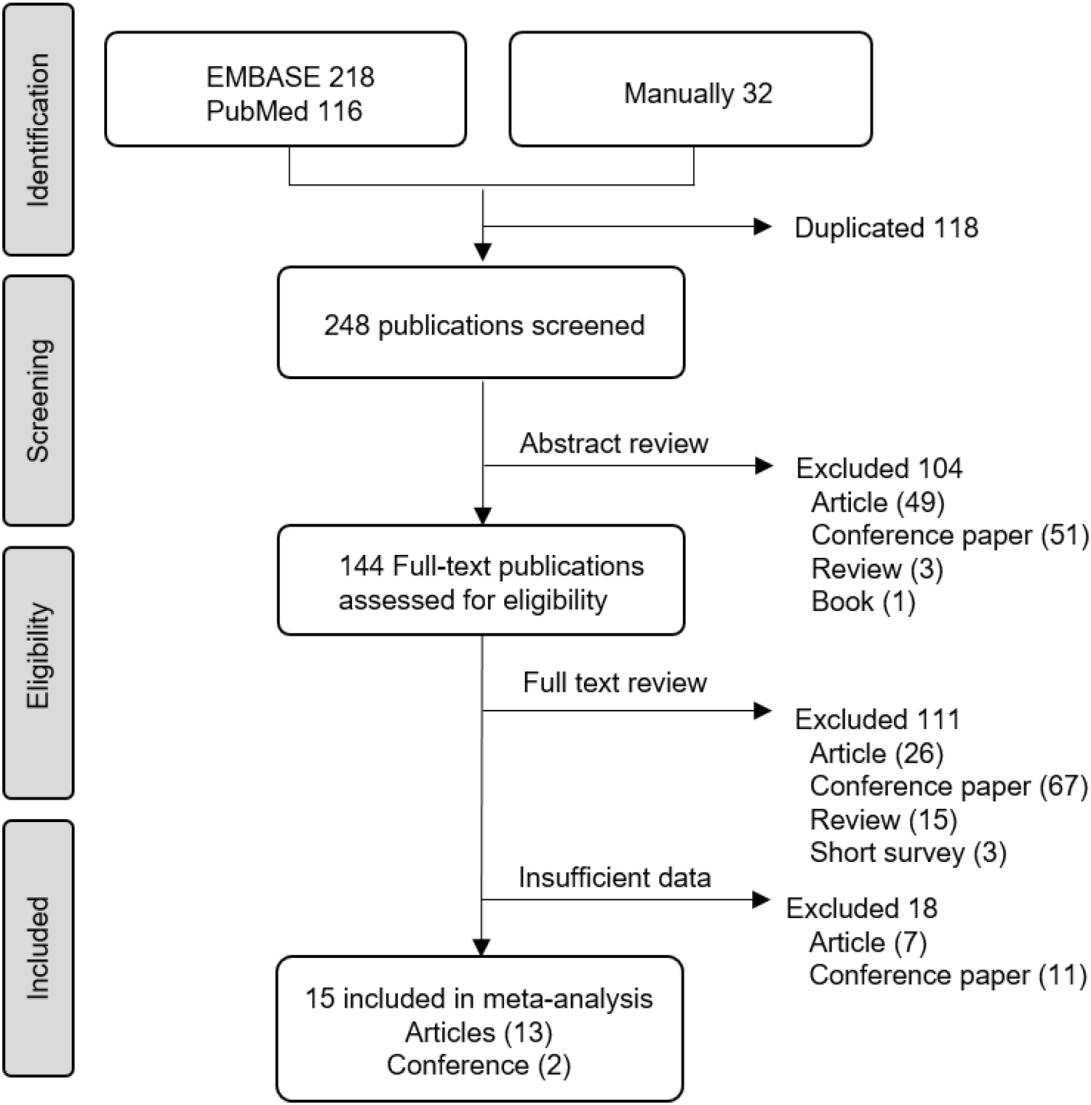
PRISMA flow diagram of study selection.

A total of 366 publications were found, with 218 from EMBASE, 116 from PubMed/MEDLINE, and 32 from manual searches (Fig. 1). After the removal of duplicates, a total of 248 remained. From reading the titles and abstracts, 144 publications judged as potentially relevant were acquired for more detailed evaluation. Another 111 publications were excluded based on the full text, as they did not meet the eligibility criteria. Subsequently, 18 publications, including 7 articles and 11 conference papers, were excluded due to insufficient data to assess the differences between anatomical and functional plans for RT therapy. Finally, a total of 309 patients with 344 RT plan sets from 15 publications were included for the meta-analysis. According to NOS scale, high and low quality studies were 13 and 2, respectively.

### Characteristics of included studies

Table 2 shows the characteristics of the included publications. The range of median ages of patients was 61–68 years. All patients were diagnosed with lung cancer of various stages. The number of included patients per study ranged from 5 to 58 (median 15, range 5–58). Seven studies had a prospective study design, and eight were retrospective studies.

**Table 2:** Basic study and patient characteristics.

Study characteristics				Patient characteristics			
First author	Year	Type of reference	Design of study	No. of patients	Age (years)	Cancer type	Stage (n)
Shioyama^19^	2007	Article	Retrospective	16	62 (median, range 40–77)	NSCLC	IIIA (3), IIIB (8), IV (3), recurrent (2)
Yin^29^	2009	Article	Prospective	10	NS	NSCLC	NS
Munawar^22^	2010	Article	Retrospective	10	NS	NSCLC	III
McGuire^18^	2010	Article	Retrospective	5	NS	Lung cancer	NS
St-Hilaire^15^	2011	Article	Retrospective	15	NS	Lung cancer	II (1), III (11), limited stage of SCLC (3)
Agrawal^17^	2012	Article	Retrospective	11	NS	NSCLC	Locally advanced
Liu^35^	2013	Conference	Retrospective	23	NS	Lung cancer	NS
Wang^23^	2013	Article	Prospective	39	61(median, range 34–77)	NSCLC	IIIA (20), IIIB (19)
Lukovic^21^	2014	Conference	Retrospective	21	NS	NSCLC	III
Meng^36^	2014	Article	Prospective	15	57–86 (range)	NSCLC	II (3), III (12)
Tian^20^	2014	Article	Retrospective	10	61 (median, range 42–80)	NSCLC	I-III
Far^3^	2015	Article	Prospective	58	67 (median, range 43–84)	NSCLC	I-II (5), III (36), IV (3), recurrent (14)
Siva^25^	2015	Article	Prospective	20	68 (median, range 47–90)	NSCLC	I (5), II (2), III (11), IV (2)
Siva^24^	2016	Article	Prospective	14	65 (median, range 47–90)	NSCLC	I (5), II (1), III (7), IV (1)
Xiao^4^	2018	Article	Prospective	42	NS	NSCLC	IIIA (19), IIIB (23)

NS non-specified; NSCLC non-small lung cancer.

### Technical aspects of nuclear imaging

Table 3 shows details of the studies including 13 SPECT and 2 PET modalities. Among 15 publications, eleven studies performed perfusion scans for RT, two studies did ventilation scans, and two studies performed both perfusion and ventilation scans. For perfusion scans, ^99m^Tc-MAA and ^68^Ga-MAA were used for SPECT and PET by intravenous injection. For ventilation scans, ^99m^Tc-labeled ultrafine graphite particles, ^99m^Tc-DTPA, and ^68^Ga-aerosol were used for SPECT and PET through inhalation.

**Table 3:** Technical aspects of nuclear imaging in the included studies.

First author	Year	Imaging type	Radiotracer (injected activity, MBq)	Type of image	Scanner	Scanning information
Shioyama^19^	2007	Q	^99m^Tc-MAA (185)	SPECT/CT	Hawkeye, GE	Pixel size 4.42 mm
Yin^29^	2009	Q	^99m^Tc-MAA (NS)	SPECT	Infinia, GE	Interval 6°, 20 s/view
Munawar^22^	2010	V	^99m^Tc-labeled ultrafine graphite particles (30)	SPECT/CT	Hawkeye, GE	NS
McGuire^18^	2011	Q	^99m^Tc-MAA (148)	SPECT	NS	Pixel size 3.56 mm Matrix 128 × 128
St-Hilaire^15^	2011	Q	^99m^Tc-MAA (185)	SPECT	E.CAM, Siemens	64 projections 15 s/view Matrix 64 × 64, 128 × 128
Agrawal^17^	2012	Q	^99m^Tc-MAA (200)	SPECT	Sofa Vision Medical DSTXL, GE	64 projections Pixel size 4.6 mm Matrix 128 × 128
Liu^35^	2013	Q	NS	SPECT	NS	NS
Wang^23^	2013	Q	^99m^Tc-MAA (200)	SPECT	Forte, Philips	NS
Lukovic^21^	2014	V	NS	SPECT	NS	NS
Meng^36^	2014	Q	^99m^Tc-MAA (185)	SPECT/CT	Symbia T6, Siemens	60 projections Interval 3°, 19 s/view Matrix 128 × 128
		V	^99m^Tc-DTPA (1,850 reservoir)	SPECT/CT	Symbia T6, Siemens	60 projections Interval 3°, 19 s/view Matrix 128 × 128
Tian^20^	2014	Q	^99m^Tc-MAA (NS)	SPECT	Forte, Phillips	Interval 6° Pixel size 4.42 mm Matrix 128 × 128
Farr^3^	2015	Q	^99m^Tc-MAA (200)	SPECT/CT	Symbia T16, Siemens	128 projections, 5 sec/view Pixel size 9.6 mm Matrix 64 × 64
Siva^25^	2015	Q	^68^Ga-MAA (40)	PET/CT	Discovery 690, GE	2 beds, 5 min/bed, respiratory-gated
		V	^68^Ga-aerosol (200 reservoir)	PET/CT	Discovery 690, GE	2 beds, 5 min/bed, respiratory-gated
Siva^24^	2016	Q	^68^Ga-MAA (40)	PET/CT	Discovery 690, GE	2 beds, 5 min/bed, respiratory-gated
Xiao^4^	2018	Q	^99m^Tc-MAA (185)	SPECT/CT	Infinia, GE	NS

*Q* perfusion, *V* ventilation, *MAA* macroaggregated albumin, *NS* non-specified.

Among the 13 studies using SPECT, only three mentioned the scanning protocols including projections, acquisition times, and matrix sizes^3,15,16^. Five studies described inadequate scanning information^17–20^, and the remaining five, including two conference papers, did not mention any information^4,21–23^. The two studies using PET provided detailed information^24,25^.

The functional images obtained from each modality were co-registered to CT planning images to provide combined datasets for RT planning. The thresholds of functional lung were variable. There was no threshold in five studies. The 30–90% threshold or the range of 20–80% of the maximum value were used for perfusion images and 30–70% of the maximum value for ventilation images. If there was at least one threshold in the study for functioning lung definition, the highest value of lung volume was selected for the meta-analysis data set. The data of no threshold in nine among 13 publications using perfusion images and in two among four publications using ventilation images were selected for meta-analysis.

### Quantifying dose to functional regions of lung

Of the 309 patients with lung cancer, DVH and DFH (dose-function histograms) were computed and compared between the two sets of plans. The definition of DFH is similar to that of DVH, in which the volume of the lung obtained from the CT images under each dose bin was replaced by the cumulative counts of voxels from the SPECT images in the DFH calculations^26,27^. The SPECT images were resampled to the same voxel dimensions as the planning CT, creating a common frame of reference to allow dose-volume calculations. All of the voxels of the SPECT images were weighted linearly according to SPECT count, and the weighting function was normalized such that its mean value averaged over all lung voxels was one^3,28^.

### Characteristics of RT planning

Table 4 shows the details of RT planning of the 15 included studies. Each study adopted various definitions of functional lung and dose-volume parameters (most included V20 and MLD). The prescribed radiation dose ranged from 40–74 Gy (mostly 60 Gy). Twelve and two studies used IMRT and 3D-CRT, respectively, and two used both modalities for the optimization of RT planning.

**Table 4:** Key findings.

First author (year)	RT planning				Functional		
	RT Planning technique	Planning software	Treatment Dose(Gy) to PTV	Dose volume parameters	Imaging type	Functioning lung threshold (% of maximum value)	Benefit of functional lung sparing
Shioyama (2007)^19^	IMRT	Pinnacle	63	V5, V10, V20, MLD	Q	None, ≥ 50%, 90%	Significant
Yin (2009)^29^	3D-CRT IMRT	Pinnacle	66	V5, V10, V20, V30, V40, MLD	Q	≥ 30%	Significant
Munawar (2010)^22^	IMRT	Pinnacle	70	MLD	V	≥ 50% or ≥ 70%	Significant
McGuire (2010)^18^	IMRT	Eclipse	40	V20, V30	Q	None	Significant
St-Hilaire (2011)^15^	IMRT	Pinnacle	45–60	V10, V20, MLD	Q	None	Significant for V10, MLD
Agrawal (2012)^17^	IMRT	ISIS 3D	60	V20, V30, MLD	Q	None	Significant
Liu (2013)^35^	IMRT	NS	NS	V20, MLD	Q	None	Significant
Wang (2013)^23^	IMRT	Pinnacle	64	V10, V15, V20, V25, V30, V35	Q	None, ≥ 30%	Significant
Lukovic (2014)^21^	IMRT	NS	70	V10, V20, MLD	V	None, ≥ 70%	Significant for ≥ 70% threshold No significance for non-threshold
Meng (2014)^36^	3D-CRT	NS	60	V20, MLD	Q	None, ≥ 30%	Significant
	3D-CRT	NS	60	V20, MLD	V	None, ≥ 30%	Significant
Tian (2014)^20^	IMRT	Pinnacle	60	V20, V30	Q	None	Significant
Farr (2015)^3^	IMRT	Eclipse	60–66	V5, V10, V20, V30, MLD	Q	20–80%	Significant for V20
Siva (2015)^25^	IMRT	Eclipse	60	V5, V10, V30, V40, V50, V60, MLD	Q	≥ 70%	Significant for V5, V10, V20 and MLD
	IMRT	Eclipse	60	V5, V10, V30, V40, V50, V60, MLD	V	≥ 50%, 70%	No significance
Siva (2016)^24^	3D-CRT	Elekta CMS/XiO	60	V5, V20, V30, V50, V60, MLD	Q	None, ≥ 30%	Significant for ≥ 30% threshold
Xiao (2018)^4^	3D-CRT IMRT	NS	60–74	V5, V10, V15, V20, MLD	Q	≥ 30%	Significant

*Q* perfusion, *V* ventilation, *PTV* planning target volume, *RT* radiotherapy, *3D-CRT* 3-dimensional conformal radiotherapy, *MLD* mean lung dose, *DFH* dose-function histogram.

Nine studies reported statistically significant benefits in terms of functional lung sparing utilizing functional imaging. One study reported no benefit, and five studies did not report benefits from functional imaging.

### Meta-analysis

The 13 articles and two conference papers were eligible for the meta-analysis. Among 15 studies, the mean ± SD data were presented in five studies, and raw data in five other studies were directly calculated as mean ± SD. The mean ± standard error in one study was converted to mean ± SD. In four studies, data were estimated from medians with lowest and highest values using the method of reference^14^.

**Figure 2:**
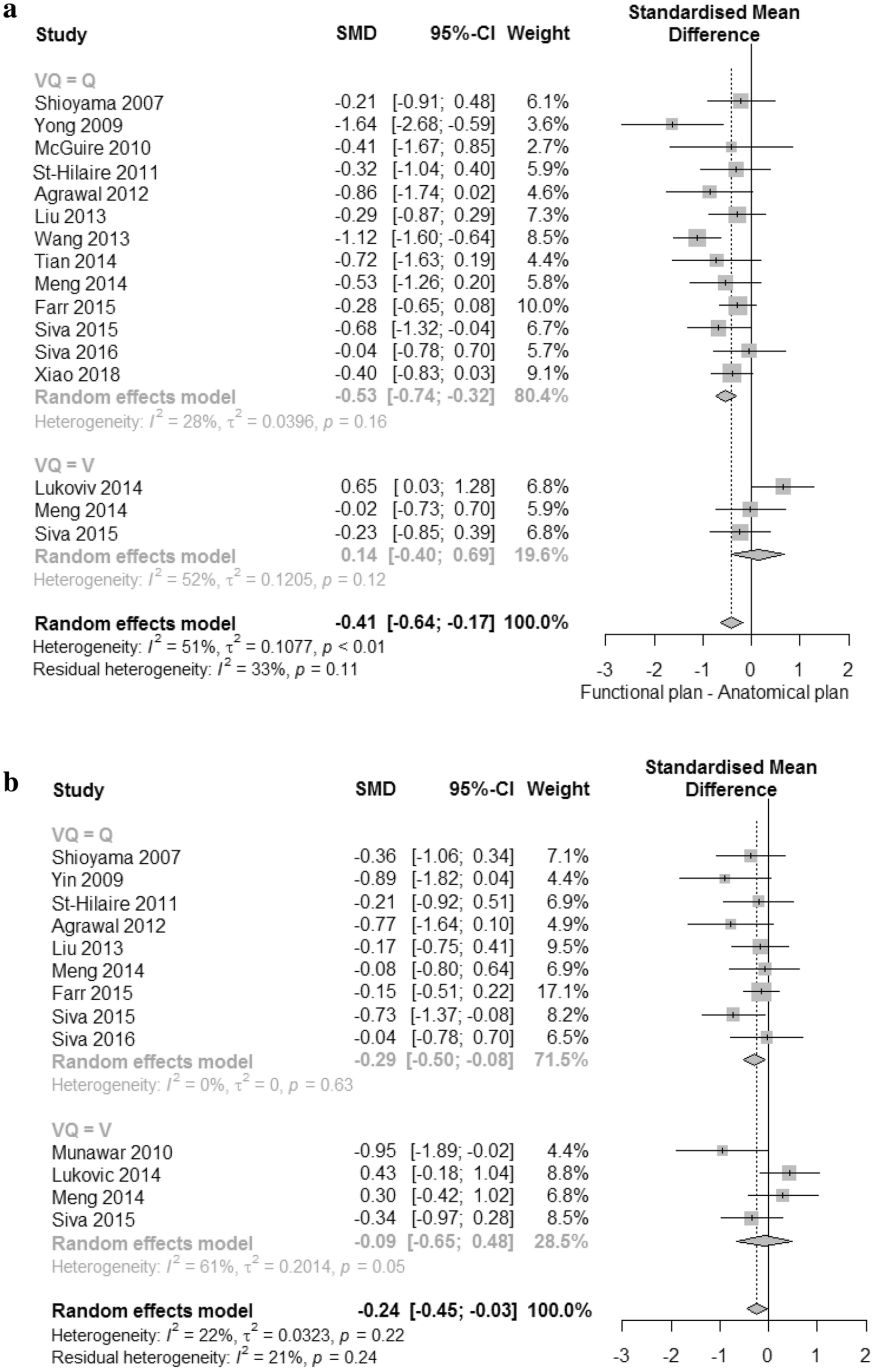

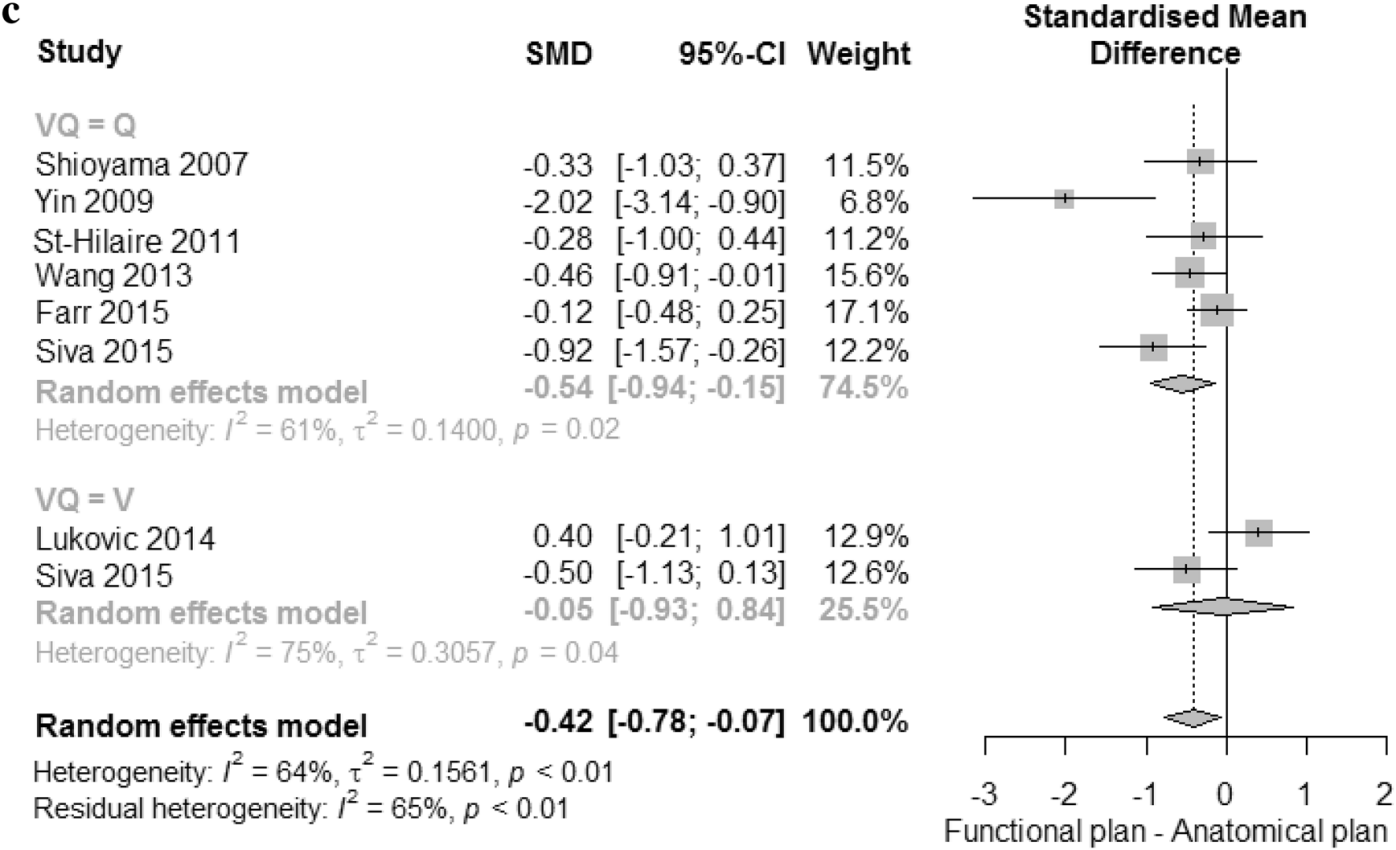
Forest plots of standard mean difference between anatomical plan and functional plan. (**a)** V20, (**b**) mean lung dose (MLD), and (**c**) V10.

The SMD in V20, MLD, and V10 between anatomical and functional plans are presented in Fig. 2. The analyses were performed separately for perfusion and ventilation images. The 14 studies were selected for V20 meta-analysis. Perfusion SPECT was performed in thirteen studies, and ventilation SPECT was done in three studies. Two studies, one each for SPECT and PET scans, performed both perfusion and ventilation scanning. For V20, the combined analysis showed significant differences between anatomical and functional plans (*P* < 0.01), and low heterogeneity (I^2^ = 33%), as shown in Fig. 2a. The standard mean in plans using perfusion images was significantly reduced by − 0.53 (95% CI − 0.74 to − 0.32); however, there was no difference between anatomical plans and functional plans using ventilation imaging (SMD 0.14 [95% CI − 0.40 to 0.69].

Funnel plots were symmetric, showing an absence of publication bias, which confirmed by trim-and-fill analysis (Supplemental Fig. 1a).

Thirteen studies were selected for the MLD meta-analysis. Two studies included both types of functional images. All other studies reported results for perfusion only (n = 7) or ventilation only (n = 2). The combined analysis showed a significant difference between anatomical and functional plans of − 0.24 (95% CI − 0.45 to − 0.03). In subgroup analysis, the fMLD of the functional plan using perfusion was significantly lower than that of the anatomical plan (MLD), a reduction of − 0.29 (95% CI − 0.50 to − 0.08), but there was no significant difference for ventilation. There was low heterogeneity within the perfusion images (I^2^ = 0%).

Seven studies were included for V10 meta-analysis. Perfusion scans were performed in six studies and ventilation scans were done in two studies. One study included both types of functional images. The significant differences were seen only in perfusion (SMD − 0.54 [95% CI − 0.94 to − 0.15]). No significant differences were found in combined or ventilation images. There was high heterogeneity both within perfusion and ventilation (I^2^ = 61% and I^2^ = 75%). Funnel plots were asymmetric, showing publication bias, which confirmed by trim-and-fill analysis for MLD and V10. (Supplemental Fig. 1b,c).

## Discussion

The current evaluation of RT planning assumes that all lung tissues function equally. However, the 15 studies included in this analysis have suggested that lung function distribution varies among lung regions by visualizing it with nuclear functional lung imaging, and this may enable functional lung avoidance in RT planning. Our results show that MLD, V20, and V10 were all improved by functional plans using nuclear images. There were significant improvements with perfusion images, but not with ventilation images.

Although our results showed that planning parameters can be improved by integrating nuclear functional imaging with RT planning, there are insufficient data regarding whether functional planning parameters could predict lung toxicity better than standard planning parameters. Only two studies included in this analysis dealt with the predictive value of functional parameters in RILI. Farr et al. showed that SPECT-based functional parameters were better to predict the risk of RILI compared to standard ones^3^. Similarly, Xiao et al. showed that SPECT-based functional parameters evaluated mid-treatment were significantly elevated in patients with RILI, and seemed to have better predictive accuracy than did the standard ones^4^. Thomas et al. recently added new evidence on usefulness of functional lung avoidance RT by reporting that increased post-treatment perfusion in low-dose regions of select patients receiving functional RT, which was not observed in any patients receiving conventional RT based on CT imaging^5^.

The effort to preserve lung function without compromising clinical outcomes is crucial for lung RT, considering lung cancer patients are usually old, and have respiratory comorbidities such as COPD or interstitial lung disease, and poor performance status. Risk-adaptive RT planning using functional lung imaging could be attempted based on our meta-analyses, especially in high-risk lung cancer patients.

### Scan modalities and protocols

The modalities and acquisition methods to obtain functional images can influence both image registration and validation between functional images and planning CT. The included publications used SPECT, SPECT/CT, or PET/CT modalities with variable scan protocols to obtain functional lung images. However, the acquisition and processing parameters (SPECT acquisition, acquisition time per projection, collimator, matrix size, reconstruction, post-reconstruction filter, etc.) within each publication were not described in detail.

Eight publications used SPECT modality, five used SPECT/CT modality, and two publications of only one group reported the functional plans using respiratory gated PET/CT. In the enrolled publications, SPECT images were performed during free breathing. The addition of CT to SPECT and PET can be accurately co-registered to CT planning images. In the cases of SPECT without CT acquisition, the markers have been proposed to be used to better match the SPECT images to the CT planning images^14,17,29^. A SPECT scan takes about 12–20 min. PET offers faster scan speeds and better image quality due to its higher sensitivity and spatial resolution compared with SPECT. A PET scan requires approximately 10 min (two bed positions at 5 min each). Since ^68^Ga-MAA PET scans in two publications were obtained under respiration-gated conditions unlike the ^99m^Tc-MAA SPECT in other publications, we did not compare the functional plans according to radiotracer differences. In addition to the acquisition methods, the differences such as patient set-up, the time interval between functional images, and planning of CT could influence registration and validation. Due to insufficient information in relation to these factors, it is difficult to discuss their implications. There is no difference in how to overlay nuclear imaging to CT scan between ventilation and perfusion SPECT or PET scan. Radiotherapy planning examples using SPECT and PET were shown in studies by St-Hilaire^15^ (Supplemental Fig. 2a) and Siva^25^ (Supplemental Fig. 2b).

### Radiotracer types

Functional images of both perfusion and ventilation are produced by different mechanisms and affected by the sizes of radiotracers. Perfusion imaging is accomplished by the microembolization of radioisotope-labeled albumin particles within pulmonary arterioles, accurately defining regional lung perfusion^30^. For perfusion imaging, each ^99m^Tc- or ^68^Ga-labeled tracer using the same carrier molecule of macroaggregated albumin (MAA) was used for perfusion SPECT and PET. PET imaging using ^68^Ga-MAA showed superior image quality as compared with ^99m^Tc-MAA SPECT imaging^31^.

For ventilation imaging, ^99m^Tc-labeled ultrafine graphite particles, ^99m^Tc-DTPA, or ^68^Ga-aerosol were used in the enrolled studies. The image quality of ventilation imaging is affected by the size of the radiotracer. The size range of ^99m^Tc-labeled ultrafine graphite particles is small at 0.005–0.2 μm^32^. The ^68^Ga-aerosol was prepared using the same technique as that for the ^99m^Tc-labeled ultrafine graphite particles with the substitution of ^68^Ga for ^99m^Tc^33^; therefore, the size of ^68^Ga-aerosol is predicted to be similar to that of the ^99m^Tc-labeled ultrafine graphite particles. The mass median aerodynamic diameter of ^99m^Tc-DTPA is 1.7 μm or less^34^, larger than the other two types. In an intra-individual comparative study of ^99m^Tc-DTPA and ^99m^Tc-labeled ultrafine graphite particles, there was a close correlation between the two tracers in patients with normal ventilation^34^. However, in patients with compromised ventilation, ^99m^Tc-DTPA had a more uneven distribution, and the risk of central and focal peripheral deposition was high. Among the four included publications using ventilation, ^99m^Tc-labeled ultrafine graphite particles, ^99m^Tc-DTPA, and ^68^Ga-aerosol were used in three different publications, while one conference publication did not describe the radiotracer used. We cautiously anticipate that if the patients have underlying airway disease, the radiotracer selection for ventilation imaging will have a tremendous impact on functional planning.

### Threshold of functional lung

The thresholds of functional lung were variable in the enrolled publications. Among 15 publications, five using perfusion images did not select a threshold for functional lung^15,17,18,20,35^. In the remaining publications, one or more thresholds (20–90% for perfusion and 30–70% for ventilation) within publications were calculated. When the threshold of perfusion was higher, fMLD and fV20 were similarly more favorable. Shioyama et al. showed that the median reductions of MLD were 2.2 Gy (17.6–14.5) and 4.2 Gy (16.0–11.8) by functional plans incorporating 50% and 90% thresholds with perfusion SPECT/CT, respectively, compared with those in the anatomic plans^19^. The other studies showed similar results^21,23,24^.

In the enrolled publications, the functional plans of ventilation had higher thresholds compared with those of perfusion. Among four studies, two studies did not improve dosimetric parameters even though a high threshold of 70 percentile was chosen^21,25^. With higher thresholds, the functional lung volumes generated have small volumes and resulted in numerous small islands throughout the lung rather than an adjacent bulk volume^3^ (See Fig. 1 in Reference^3^). The appropriate threshold of functional lung has been evaluated in the related literature; however, there is no clinically optimal threshold to determine the functional lung in both perfusion and ventilation images, and the threshold value is likely to vary depending on the imaging type.

We excluded all studies where the mean ± SD of both anatomical and functional plans were not mentioned as a specific value or where median values with confidence intervals were not provided. Many of the excluded studies were related to perfusion or ventilation SPECT to observe changes in functional lung before and after radiation therapy. Besides nuclear medicine imaging, CT and MRI are other modalities used in attempts to obtain a functional ventilation image. There are few studies on the uses of functional CT and MRI for radiation planning in patients with lung cancer. CT ventilation had variables in technique and MRI seems to be still in the trial phase.

Our study had limitations. There was no randomized interventional study, and all included studies had small numbers of patients. Our meta-analysis demonstrated that the MLD, V20, and V10 were improved by RT planning using nuclear functional images in patients with lung cancer. The results of the study show that only perfusion is significantly useful. It is difficult to conclude that ventilation imaging is not useful for RT planning, because there were only a few related studies. Future studies on this topic are needed.

## Supplementary information

Supplementary Information.
